# Bayesian estimation of direct and correlated responses to selection on linear or ratio expressions of feed efficiency in pigs

**DOI:** 10.1186/s12711-018-0403-0

**Published:** 2018-06-20

**Authors:** Mahmoud Shirali, Patrick Francis Varley, Just Jensen

**Affiliations:** 1 Center for Quantitative Genetics and Genomics, Department of Molecular Biology and Genetics, Aarhus University, 8830 Tjele, Denmark; 2 Hermitage Genetics, Kilkenny, Ireland

## Abstract

**Background:**

This study aimed at (1) deriving Bayesian methods to predict breeding values for ratio (i.e. feed conversion ratio; FCR) or linear (i.e. residual feed intake; RFI) traits; (2) estimating genetic parameters for average daily feed consumption (ADFI), average daily weight gain (ADG), lean meat percentage (LMP) along with the derived traits of RFI and FCR; and (3) deriving Bayesian estimates of direct and correlated responses to selection on RFI, FCR, ADG, ADFI, and LMP. Response to selection was defined as the difference in additive genetic mean of the selected top individuals, expected to be parents of the next generation, and the total population after integrating genetic trends out of the posterior distribution of selection responses. Inferences were based on marginal posterior distributions obtained from the Bayesian method for integration over unknown population parameters and “fixed” environmental effects and for appropriate handling of ratio traits. Terminal line pigs (n = 3724) were used for a multi-variate model for ADFI, ADG, and LMP. RFI was estimated from the conditional distribution of ADFI given ADG and LMP, using either genetic (RFI_G_) or phenotypic (RFI_P_) partial regression coefficients. The posterior distribution of the FCR’s breeding values was derived from the posterior distribution of “fixed” environmental effects and additive genetic effects on ADFI and ADG.

**Results:**

Posterior means of heritability were 0.32, 0.26, 0.56, 0.20, and 0.15 for ADFI, ADG, LMP, RFI_P_, and RFI_G_, respectively. Selection against RFI_G_ showed a direct response of − 0.16 kg/d and correlated responses of − 0.16 kg/kg for FCR and − 0.15 kg/d for ADFI, with no effect on other production traits. Selection against FCR resulted in a direct response of − 0.17 kg/kg and correlated responses of − 0.14 kg/d for RFI_G_, − 0.18 kg/d for ADFI, and 0.98% for LMP.

**Conclusions:**

The Bayesian methodology developed here enables prediction of breeding values for FCR and RFI from a single multi-variate model. In addition, we derived posterior distributions of direct and correlated responses to selection. Genetic parameter estimates indicated a genetic basis for the studied traits and that genetic improvement through selection was possible. Direct selection against FCR or RFI_P_ resulted in unexpected responses in production traits.

## Background

In swine breeding programs, efficiency of nutrient use is a significant factor because of its economic and environmental importance. Classically, feed efficiency is defined as output over input, for instance, milk yield, or milk components yield over dry matter consumption in dairy cattle, or body weight gain over feed consumption in pigs. However, in swine breeding programs, feed conversion ratio (FCR) is primarily used, defined as average daily feed intake (ADFI) over average daily body weight gain (ADG).

The distribution of ratio traits such as FCR depends on the joint distribution of two normally distributed variables. The distribution of a ratio trait is easily determined if the mean of each random variable and the correlation between them are equal to zero. However, complexity arises as the variables’ means and correlation deviate from zero [1, 2]. The ratio of two correlated normal random variables has a closed approximate form, as illustrated by Hinkley [2], and a distribution that deviates from normality, as reported by Gunsett [3]. Therefore, selection for ratio traits often results in unexpected responses in its component traits [3].

To circumvent the problems of ratio traits, residual feed intake (RFI) was proposed by Koch et al. [4] as a better measure to determine animal feed efficiency. RFI is a partial measure of feed efficiency that refers to the proportion of feed intake that is independent of performance. In the classical definition, RFI is observed as ADFI minus the expected ADFI based on body weight (BW) and ADG, along with carcass composition, e.g., lean meat percentage (LMP), based on the results of a multiple regression analysis. Following Kennedy et al. [5], this could be termed phenotypic RFI, as the correction ensures that the phenotypic covariance between RFI and production traits (i.e. BW, ADG and LMP) is zero. If the genetic (co)variances for the component traits of RFI (e.g., ADFI, ADG and LMP) are known, a genetic RFI can be computed using partial genetic regression coefficients of ADFI on production traits (e.g., ADG and LMP), as applied by Shirali et al. [6]. Using a Bayesian framework, Jensen [7] showed that breeding values and the posterior distribution of RFI can be derived by defining the proper distributions of feed intake, conditional on BW and ADG, and potentially other traits that act as important energy sinks, such as body fat content. This procedure also circumvents the need for deriving the regression coefficients from a separate regression analysis first and then using them in genetic analysis to compute a phenotypic RFI. Using a multivariate animal model can ensure that parameter estimation in the regression analysis is not biased by fixed effects in the model, or by effects due to genetic trends for component traits in the population under investigation [7].

Bayesian methodology, as illustrated by Sorensen et al. [8], provides marginal posterior distributions for any parameter in the model, given the data available, where the required posterior distributions are obtained by means of the Gibbs sampler [9]. If non-informative priors are used, these distributions consider that other parameters, such as the variance components, are inferred from the data, such that proper probability statements can be made for response to selection. Bayesian methods ensure that uncertainties about the fixed effects and variance components are considered when evaluating breeding values and estimates of responses to selection. The Bayesian approach allows inference of the posterior distributions of non-linear functions of parameters, even if their distributions are unknown. Genetic or phenotypic variances and covariances in a given generation can be inferred based on their marginal posterior distributions, as shown by Sorensen et al. [10] for a univariate model. Inferences about breeding values are made using the marginal posterior distribution of the vector of breeding values. Marginal posterior distributions of responses to selection or of genetic superiorities of a selected group can be obtained by averaging predicted breeding values that are obtained using mixed model techniques, as shown by Sorensen et al. [8]. In addition, when variance components are known and flat priors are used for fixed effects, the Bayesian estimates of response to selection are identical to the analysis by Sorensen and Kennedy [11].

The aims of this study were to (1) derive methods for the Bayesian prediction of breeding values for phenotypic and genetic RFI and for FCR, without invoking unrealistic distributional assumptions for FCR; (2) estimate genetic parameters for the production traits of growth, feed intake, and lean meat production, and for the derived traits of RFI and FCR; and (3) derive Bayesian estimates of direct and correlated responses to selection for feed efficiency, measured either as RFI or FCR, and for production traits.

## Methods

### Data

Animal care and handling were performed as part of a routine commercial breeding program. Animals were reared using standard procedures in a commercial Irish pig farm and therefore, no further approval of animal care and handling procedures was necessary. The dataset used for this study was collected as routine feed intake records from 2007 to 2014. Pigs (n = 3027; 2621 boars and 406 gilts) originated from Hermitage Genetics (Kilkenny, Ireland) and were selected on an index comprising feed conversion ratio, days to achieve 110 kg, and lean meat percentage (LMP). Animals went on trial at 52 kg (11, SD) and daily feed intake records were collected until they reached 110 kg (10, SD) of BW. During the test period, pigs were kept in mixed-sex pens of 12 pigs each, equipped with IVOG electronic feeders (Insentec B.V., Marknesse, The Netherlands). Pig were fed ad libitum using a standard wheat and barley-based Irish finisher diet with 13.7 megajoules of digestible energy, 17% crude protein, and 0.97% standard ileal digestible lysine per kg of feed. The test period lasted a maximum of 8 weeks. Raw data contained records from each entry to the feeder during the test period. Feed intake errors in single visits to the feeding station were identified following the algorithm developed by Casey et al. [12] and were removed from the dataset. Descriptive statistics for the data are in Table 1. ADFI was calculated as total feed intake in the entire test period, divided by the number of days on test. ADG was calculated as total body weight gain divided by the number of days on test. LMP was predicted using a transformation of fat layer and muscle depths between the 3rd and 4th last ribs from ultrasound images taken at the end of the test period using a Piglog 105 ultrasonic device (Carometec A/S, Denmark). Pedigree information was available for at least the last four generations, for 6237 animals.

**Table 1:** Mean and standard deviation (SD) of average daily feed intake (ADFI), average daily gain (ADG), lean meat percentage (LMP), and start (SBW) and end body weight (EBW)

Trait	Mean	SD
ADFI, kg/d	2.61	0.39
ADG, kg/d	1.12	0.15
LMP, %	62.7	1.97
SBW, kg	54.9	11.3
EBW, kg	109	10.3

### Statistical models

Tri-variate analysis was used for ADFI, ADG, and LMP traits using the following models:

1 $${\mathbf{y}}_{\text{ADFI}} = {\mathbf{Xb}}_{\text{ADFI}} + b_{ADFI} {\mathbf{x}}_{\text{s}} + {\mathbf{Za}}_{\text{ADFI}} + {\mathbf{Sp}}_{\text{ADFI}} + {\mathbf{e}}_{\text{ADFI}} ,$$

2 $${\mathbf{y}}_{\text{ADG}} = {\mathbf{Xb}}_{\text{ADG}} + b_{ADG} {\mathbf{x}}_{\text{s}} + {\mathbf{Za}}_{\text{ADG}} + {\mathbf{Sp}}_{\text{ADG}} + {\mathbf{e}}_{\text{ADG}} ,$$

3 $${\mathbf{y}}_{\text{LMP}} = {\mathbf{Xb}}_{\text{LMP}} + b_{LMP} {\mathbf{x}}_{\text{e}} + {\mathbf{Za}}_{\text{LMP}} + {\mathbf{Sp}}_{\text{LMP}} + {\mathbf{e}}_{\text{LMP}} ,$$

where **y**_ADFI_, **y**_ADG_ and **y**_LMP_ are vectors of phenotypic records for ADFI, ADG, and LMP, respectively; vectors **b**_ADFI_, **b**_ADG_, and **b**_LMP_ contain “fixed” effects of year-quarter, gender, and sow parity for ADFI, ADG and LMP, respectively; *b*_*ADFI*_ and *b*_*ADG*_ are “fixed” regressions for start body weight for ADFI and ADG, respectively; *b*_*LMP*_ is the “fixed” regression for end body weight for LMP; **a**_ADFI_, **a**_ADG_, and **a**_LMP_, **p**_ADFI_, **p**_ADG_, and **p**_LMP_, **e**_ADFI_, **e**_ADG_, and **e**_LMP_ are vectors of animal additive genetic, pen, and residual effects for ADFI, ADG, and LMP, respectively. The permanent environment effect of litter of origin had a small effect based on an initial likelihood ratio test and therefore was not included in the model. Matrices **X** are design matrices for year-quarter, gender, and parity effects; **x**_s_ is a vector of start body weights for each animal and **x**_e_ is a vector of end body weights. Matrices **Z** and **S** are the corresponding design matrices for additive genetic animal effects (**a**_ADFI_, **a**_ADG_, and **a**_LMP_) and the permanent environmental effect of pen (**p**_ADFI_, **p**_ADG_, and **p**_LMP_) for the three traits. Average BW was not included in the model because animals were tested over a fixed weight interval; therefore, all animals had the same average weight. A full Bayesian analysis was conducted and, therefore, priors were specified for all parameters. Prior distributions for all random vectors were multivariate normal distributions with a mean of zero, and $${\text{Var}}\left( {\begin{array}{*{20}c} {\begin{array}{*{20}c} {{\mathbf{e}}_{\text{ADFI}} } \\ {{\mathbf{e}}_{\text{ADG}} } \\ \end{array} } \\ {{\mathbf{e}}_{\text{LMP}} } \\ \end{array} } \right) = {\mathbf{I}} \otimes {\mathbf{R}}_{0}$$, where **R**_0_ is a 3 × 3 matrix of residual (co)variances, $${\text{Var}}\left( {\begin{array}{*{20}c} {\begin{array}{*{20}c} {{\mathbf{a}}_{\text{ADFI}} } \\ {{\mathbf{a}}_{\text{ADG}} } \\ \end{array} } \\ {{\mathbf{a}}_{\text{LMP}} } \\ \end{array} } \right) = {\mathbf{A}} \otimes {\mathbf{G}}_{0}$$, where **A** is the additive genetic relationship matrix, **G**_0_ is a 3 × 3 matrix of additive genetic (co)variances, and genetic values are ordered by individual; and $${\text{Var}}\left( {\begin{array}{*{20}c} {\begin{array}{*{20}c} {{\mathbf{p}}_{\text{ADFI}} } \\ {{\mathbf{p}}_{\text{ADG}} } \\ \end{array} } \\ {{\mathbf{p}}_{\text{LMP}} } \\ \end{array} } \right) = {\mathbf{I}} \otimes {\mathbf{K}}_{0}$$, where **K**_0_ is a 3 × 3 matrix of pen (co)variances. The random effects of **a**, **p**, and **e** were considered independent of each other. The prior distributions for the covariance matrices **G**_0_, **K**_0_, and **R**_0_ were inverse Wishart distributions and priors for all dispersion and for all “fixed” location parameters were taken as flat priors.

The Bayesian estimation method via Gibbs sampling was used to obtain posterior distributions for all parameters that were included in the trivariate models (1), (2), and (3), including the matrices of variances and covariances. The Gibbs sampler was run for 1.1 million rounds, with the first 100,000 rounds considered burn-in, and after the burn-in, every 250th sample was saved for posterior analysis. The RJMC module in the DMU software package by Madsen and Jensen [13] was used for analysis.

### Analysis of posterior distributions

A total of 4000 samples from the joint posterior distribution of all location and (co)variance parameters from the trivariate models (1)–(3) were saved for post-Gibbs analysis. The BOA package of Smith [14] in the R program [15] was used for convergence diagnostics through statistical and graphical analysis of the posterior distributions of the (co)variance, location, and derived parameters. The results indicated convergence of all parameters investigated.

Let **s**_*i*_ be the vector of all model parameters in sample *i* from the marginal posterior distribution of **s**. Any feature or function of the distribution can be obtained using the ergodic theorem shown by Geyer [16] and Smith and Roberts [9]:

4 $$\hat{\mu }_{m} = \frac{1}{m}\mathop \sum \limits_{i = 1}^{m} g\left( {{\mathbf{s}}_{i} } \right) ,$$

where *g*(.) is an appropriate operator, *μ* is any function or feature of the marginal distribution of **s**, and *m* is the number of samples obtained. For more details on how to use this to estimate response in selection experiments, see Sorensen et al. [8]. Then, we derived functions to define the posterior distribution of genetic and residual (co)variances to derive breeding values for various RFI and FCR definitions. In addition, functions to infer the amount of genetic variance and covariance available for selection were derived, along with responses to selection or of genetic superiorities of the selected groups for different selection criteria. Functions to define posterior genetic and residual variances for FCR are not available without resorting to Taylor series approximations but the amount of genetic variance in FCR available for selection can be derived from the output of the Gibbs sampler without resorting to approximations. The functions to predict RFI and FCR were used in every sample obtained and Eq. (4) was used to obtain summary information on the distribution of this function, i.e. the posterior mean and variance of genetic variance.

### Posterior distribution of RFI

RFI was defined as (1) phenotypic RFI (RFI_P_) using phenotypic partial regression coefficients to ensure that phenotypic covariances are zero; and (2) genetic RFI (RFI_G_), conditioning breeding values of ADFI by breeding values for ADG and LMP using genetic partial regression coefficients, ensuring that the genetic covariances between RFI_G_ and production traits (ADG and LMP) are zero. In other words, the breeding values for ADFI are corrected for ADG and LMP using either genetic or phenotypic regression coefficients. In this section, we present the derivation of variance parameters and breeding values for both RFI forms, which are both linear combinations of the traits included in the analysis. In each individual sample (***s***), derivation of both the distribution and the breeding values of the RFI traits are straightforward because they are conditional on the (co)variance components, and all elements are from multivariate normal distributions. These derivations are used on all samples obtained from the Gibbs sampler to obtain the posterior distributions of (co)variances and breeding values for the two RFI definitions. Across the posterior samples, the distribution is, however, not necessarily normal.

For RFI_G_, the partial regression coefficients (**b**_G_) for ADG and LMP were computed from the genetic (co)variance matrix, while for RFI_P_ the partial phenotypic regression coefficients (**b**_P_) were from the phenotypic (co)variance matrix. Within a posterior sample, both RFI definitions involved conditional normal distributions, resulting in the following straightforward derivations: let ${\mathbf{P}}_{0} = {\mathbf{G}}_{0} + {\mathbf{K}}_{0} + {\mathbf{R}}_{0}$ be the phenotypic and **G**_0_ the genetic (co)variance matrices of the traits involved, which are subdivided in:

$${\mathbf{P}}_{0} = \left[ {\begin{array}{*{20}c} {{\text{P}}_{ADFI} } & {{\text{P}}_{ADFI,ADG} } & {{\text{P}}_{ADFI,LMP} } \\ {{\text{P}}_{ADG,ADFI} } & {{\text{P}}_{ADG} } & {{\text{P}}_{ADG,LMP} } \\ {{\text{P}}_{LMP,ADFI} } & {{\text{P}}_{LMP,ADG} } & {{\text{P}}_{LMP} } \\ \end{array} } \right]\,{\text{and}}\quad {\mathbf{G}}_{0} = \left[ {\begin{array}{*{20}c} {{\text{G}}_{ADFI} } & {{\text{G}}_{ADFI,ADG} } & {{\text{G}}_{ADFI,LMP} } \\ {{\text{G}}_{ADG,ADFI} } & {{\text{G}}_{ADG} } & {{\text{G}}_{ADG,LMP} } \\ {{\text{G}}_{LMP,ADFI} } & {{\text{G}}_{LMP,ADG} } & {{\text{G}}_{LMP} } \\ \end{array} } \right] ,$$

where the diagonals of matrices are the variances and the off-diagonals are the covariances.

Bayesian estimation of partial phenotypic (**b**_P_) and genetic (**b**_G_) regression coefficients was obtained as:

5 $${\mathbf{b}}_{\text{P}} = {\mathbf{P}}_{\text{p}}^{ - 1} {\mathbf{P}}_{{{\text{p}},{\text{ADFI}}}} \quad {\text{and}}\quad {\mathbf{b}}_{\text{G}} = {\mathbf{G}}_{\text{p}}^{ - 1} {\mathbf{G}}_{{{\text{p}},{\text{ADFI}}}} ,$$

which are 2 × 1 vector-valued functions that are obtained in each sample from the Gibbs output. The **P**_p_ and **G**_p_ are 2 × 2 matrices of phenotypic and genetic (co)variance for the production traits of ADG and LMP from **P**_0_ and **G**_0_, respectively. Matrices **P**_p,ADFI_ and **G**_p,ADFI_ are the phenotypic and genetic covariances, respectively, of the production traits ADG and LMP with ADFI.

Predictions of breeding values for RFI can be obtained simultaneously for all animals by the distribution of breeding values for ADFI (**a**_ADFI_), conditional of breeding values for ADG (**a**_ADG_) and LMP (**a**_LMP_), using either phenotypic (**b**_P_) or genetic (**b**_G_) partial regression coefficients. A sample from the posterior distribution of breeding values for phenotypic (${\mathbf{a}}_{{{\text{RFI}}_{\text{P}} }}$) and genetic (${\mathbf{a}}_{{{\text{RFI}}_{\text{G}} }}$) RFI is as follows:

6 $${\mathbf{a}}_{{{\text{RFI}}_{\text{P}} }} = {\mathbf{a}}_{\text{ADFI}} - \left[ {\begin{array}{*{20}c} {{\mathbf{a}}_{\text{ADG}} } & {{\mathbf{a}}_{\text{LMP}} } \\ \end{array} } \right]{\mathbf{b}}_{\text{P}} ,$$

7 $${\mathbf{a}}_{{{\text{RFI}}_{\text{G}} }} = {\mathbf{a}}_{\text{ADFI}} - \left[ {\begin{array}{*{20}c} {{\mathbf{a}}_{\text{ADG}} } & {{\mathbf{a}}_{\text{LMP}} } \\ \end{array} } \right]{\mathbf{b}}_{\text{G}} .$$

For a given sample in ***s***_*i*_, distributions of RFI were obtained as the distribution of ADFI conditional on all other model parameters and on ADG and LMP. The corresponding variances and covariances can be obtained using the following equations:

8 $$\left[ {\begin{array}{*{20}c} {var\left( {g_{ADFI} } \right)} & {} & {} & {} & {covariance} \\ {} & {var\left( {g_{{RFI_{P} }} } \right)} & {} & {} & {} \\ {} & {} & {var\left( {g_{{RFI_{G} }} } \right)} & {} & {} \\ {} & {} & {} & {var\left( {g_{ADG} } \right)} & {} \\ {covariance} & {} & {} & {} & {var\left( {g_{LMP} } \right)} \\ \end{array} } \right] = {\mathbf{B}} {\mathbf{G}}_{0} {\mathbf{B^{\prime}}} ,$$

9 $$\left[ {\begin{array}{*{20}c} {var\left( {p_{ADFI} } \right)} & {} & {} & {} & {covariance} \\ {} & {var\left( {p_{{RFI_{P} }} } \right)} & {} & {} & {} \\ {} & {} & {var\left( {p_{{RFI_{G} }} } \right)} & {} & {} \\ {} & {} & {} & {var\left( {p_{ADG} } \right)} & {} \\ {covariance} & {} & {} & {} & {var\left( {p_{LMP} } \right)} \\ \end{array} } \right] = {\mathbf{B}} {\mathbf{P}}_{0} {\mathbf{B}}' ,$$

where ${\mathbf{B}} {\mathbf{G}}_{0} {\mathbf{B^{\prime}}}$ and ${\mathbf{B}} {\mathbf{P}}_{0} {\mathbf{B}}'$ are genetic or phenotypic (co)variances, respectively.

$${\mathbf{B}} = \left[ {\begin{array}{*{20}c} 1 & 0 & 0 \\ 1 & { - {\text{b}}_{{{\text{P}},{\text{ADG}}}} } & { - {\text{b}}_{{{\text{P}},{\text{LMP}}}} } \\ 1 & { - {\text{b}}_{{{\text{G}},{\text{ADG}}}} } & { - {\text{b}}_{{{\text{G}},{\text{LMP}}}} } \\ 0 & 1 & 0 \\ 0 & 0 & 1 \\ \end{array} } \right] ,$$

where ${\text{b}}_{{{\text{P}},{\text{ADG}}}}$ and ${\text{b}}_{{{\text{P}},{\text{LMP}}}}$ are phenotypic partial regression coefficients from ${\mathbf{b}}_{\text{P}}$, and ${\text{b}}_{{{\text{G}},{\text{ADG}}}}$ and ${\text{b}}_{{{\text{G}},{\text{LMP}}}}$ are genetic regression coefficients from ${\mathbf{b}}_{\text{G}}$ for ADG and LMP, respectively.

### Posterior distribution of FCR

FCR is a ratio between two normally distributed and usually correlated traits and therefore has a distribution that depends on the means of the two traits involved, as well as their (co)variance. As a result, the breeding value for FCR depends on “fixed” location parameters, since it depends on the mean of ADFI ($\upmu_{\text{ADFI}}$) and ADG ($\upmu_{\text{ADG}}$). Following Gunsett [3], the breeding value for FCR (${\mathbf{a}}_{\text{FCR}}$) can be calculated from underlying parameters using the following equation for a given sample $\varvec{s}_{i}$:

10 $${\mathbf{a}}_{\text{FCR}} = \frac{{\upmu_{\text{ADFI}} + {\mathbf{a}}_{\text{ADFI}} }}{{\upmu_{\text{ADG}} + {\mathbf{a}}_{\text{ADG}} }} - \frac{{\upmu_{\text{ADFI}} }}{{\upmu_{\text{ADG}} }} ,$$

where the estimate of $\upmu_{\text{ADFI}}$ can be obtained from Model (1) for ADFI as the sum of the average of each “fixed” effect (year-quarter, gender, and parity), in addition to “fixed” regressions for the start BW according to the population average. Similarly, an estimate of $\upmu_{\text{ADG}}$ can be obtained. Location parameters for the mean must be computed once per sample as we investigate functions of the variables in the posterior distribution and are applied to Eq. (10) to compute breeding values. In this way, the inaccuracy of computing the mean is considered when deriving the posterior distribution of breeding values for FCR.

Correspondingly, the phenotypic deviation of FCR can be expressed as:

11 $${\mathbf{p}}_{\text{FCR}} = \frac{{\upmu_{\text{ADFI}} + {\mathbf{a}}_{\text{ADFI}} + {\mathbf{e}}_{\text{ADFI}} }}{{\upmu_{\text{ADG}} + {\mathbf{a}}_{\text{ADG}} + {\mathbf{e}}_{\text{ADG}} }} - \frac{{\upmu_{\text{ADFI}} }}{{\upmu_{\text{ADG}} }} .$$

However, this expression cannot be used directly to derive the phenotypic variance of FCR due to influences such as selection and genetic drift. Instead, it can be used to compute the phenotypic variance of the derived traits (FCR, ${\text{RFI}}_{\text{G}}$, and ${\text{RFI}}_{\text{P}}$) and the recorded traits (ADFI, ADG, and LMP) between animals that have phenotypic records and are available for selection.

### Genetic trends

Genetic trends are defined as a linear function of the vector of breeding values following Sorensen et al. [8]:

12 $${\mathbf{r}}_{j} = \left( {{\mathbf{T^{\prime}T}}} \right)^{ - 1} {\mathbf{T}}'{\mathbf{a}}_{j} ,$$

where ${\mathbf{a}}_{\varvec{j}}$ is the vector of breeding values for trait ${\mathbf{j}},\varvec{ }\left( {{\mathbf{j}} = {\mathbf{ADFI}},{\mathbf{ADG}}, \ldots ,{\mathbf{FCR}}} \right)$; ${\mathbf{r}}_{\varvec{j}}$ is a vector of yearly means of breeding values, and ${\mathbf{T}}$ is an incidence matrix relating the breeding values of individuals to yearly batches.

### Genetic (co)variance available for selection and direct and correlated responses to selection

Since genetic selection is usually performed within an age group, the amount of genetic (co)variance available for selection at a given time point is:

13 $${\mathbf{G}}_{0}^{*} = Var\left( {\left[ {\begin{array}{*{20}c} {\left( {{\mathbf{a}}_{\text{ADFI}} - {\mathbf{Tr}}_{\text{ADFI}} } \right),} & {\left( {{\mathbf{a}}_{\text{ADG}} - {\mathbf{Tr}}_{\text{ADG}} } \right),} & \ldots & {\left( {{\mathbf{a}}_{\text{FCR}} - {\mathbf{Tr}}_{\text{FCR}} } \right)} \\ \end{array} } \right]} \right) ,$$

where ${\mathbf{G}}_{0}^{*}$ is the distribution of genetic (co)variance available for selection after integrating over the genetic trend and **T** and **r** $\left( {{\mathbf{j}} = {\mathbf{ADFI}}, {\mathbf{ADG}}, \ldots ,{\mathbf{FCR}}} \right)$ were defined in Eq. (12). This derivation is an extension of Sorensen et al. [10] to a multivariate setting.

The Bayesian estimate of the superiority of a selected group is the difference between the mean of the breeding values in the selected group and the mean of the breeding values of all animals corrected for the genetic trend. This yields an expression of the superiority of the selected group in every sample from the posterior distribution, depending on the selection rule.

The mean of the selected group for trait *j* when selecting on trait *j*^′^ can be calculated as:

14 $${\bar{\text{a}}}_{{jj^{ '} }}^{\text{s}} = \frac{1}{{n_{s} }}\mathop \sum \limits_{i = 1}^{n} {\text{a}}_{ij}^{ *} {\text{I}}({\text{a}}_{{ij^{\prime}}}^{ *} > {\text{a}}_{{n_{s} j^{'} }}^{ *} ) ,$$

where ${\text{a}}_{ij}^{ *}$ is the breeding value for trait *j* on animal *i*, conditional on the genetic trend; *n* is the total number of animals; and ${\text{a}}_{{n_{s} j^{'} }}^{ *}$ is the breeding value for a ranked individual (*n*_*s*_) when ordering breeding values for trait *j*^′^. If $j = j^{'}$, the superiority is due to direct selection for the trait, and if $j \ne j^{'}$, the superiority is in trait $j$ due to selection on a correlated trait *j*^′^. Six traits were investigated in this study and thus, six scenarios were developed to compare direct and correlated responses to selection for feed efficiency and production traits. The number of individuals ranked for analysis was decided based on truncation selection of the top 5 to 30% of animals. Here, only the results of truncation selection of the top 10% are presented, since the results and conclusions were consistent across various truncation selection percentages.

## Results

### Genetic parameters of production and feed efficiency traits

Posterior means and standard deviations (PSD) of heritability and genetic variances for the two ${\text{RFI}}$ definitions and their component traits are in Table 2. The posterior mean of heritability was moderately high for the production traits ADFI and ADG and high for LMP. The posterior means of heritability and genetic variance were larger for ADFI than for ADG. For linear feed efficiency traits, the posterior means of heritability were low for ${\text{RFI}}_{\text{G}}$ and moderate for ${\text{RFI}}_{\text{P}}$ because of a lower posterior mean of genetic variance for ${\text{RFI}}_{\text{G}}$ compared to ${\text{RFI}}_{\text{P}}$.

**Table 2:** Posterior means of heritability (h^2^) and genetic variance (${\varvec{\upsigma}}_{{\mathbf{A}}}^{2}$) of average daily feed intake (ADFI), average daily gain (ADG), lean meat percentage (LMP), and phenotypic and genetic residual feed intake (${\mathbf{RFI}}_{{\mathbf{P}}}$ and ${\mathbf{RFI}}_{{\mathbf{G}}}$, respectively), with posterior standard deviations in parentheses

Traits	ADFI, kg/d	ADG, kg/d	LMP, %	RFI_P_, kg/d	RFI_G_, kg/d
h^2^	0.32 (0.04)	0.26 (0.04)	0.56 (0.06)	0.20 (0.03)	0.15 (0.03)
$\upsigma_{\text{A}}^{2}$	0.035 (0.005)	0.004 (0.001)	2.024 (0.252)	0.011 (0.002)	0.009 (0.002)

Posterior means (with PSD) of genetic and phenotypic correlations for the two ${\text{RFI}}$ definitions and their component traits are in Table 3. As ${\text{RFI}}_{\text{G}}$ was defined using genetic partial regression coefficients, its genetic correlations with the production traits ADG and LMP were zero. The posterior mean of genetic correlation of ${\text{RFI}}_{\text{P}}$ was positive and moderate with ADG (0.35) and negative and low with LMP (− 0.06). Since partial phenotypic coefficients were used, posterior means of phenotypic correlations of ${\text{RFI}}_{\text{p}}$ with ADG and LMP were zero. Posterior means of the genetic correlation were strong and positive between ADFI and ADG (0.82) and moderate and negative between ADFI and LMP (− 0.39). The posterior mean of the genetic correlation between ADFI and ${\text{RFI}}_{\text{G}}$ was 0.51 but was larger between ADFI and ${\text{RFI}}_{\text{P}}$, 0.77.

**Table 3:** Posterior means of genetic (below diagonal) and phenotypic correlations (above diagonal) of genetic and phenotypic residual feed intake (**RFI**_**G**_ and **RFI**_**P**_, respectively), average daily feed intake (ADFI), average daily gain (ADG), and lean meat percentage (LMP), with posterior standard deviations in parentheses

Traits	RFI_G_	RFI_P_	ADFI	ADG	LMP
RFI_G_		0.95 (0.03)	0.47 (0.08)	− 0.30 (0.08)	0.05 (0.05)
RFI_P_	0.92 (0.04)		0.71 (0.01)	0.00 (0.00)	0.00 (0.00)
ADFI	0.51 (0.05)	0.77 (0.04)		0.68 (0.01)	− 0.30 (0.02)
ADG	0.00 (0.00)	0.35 (0.10)	0.82 (0.04)		− 0.17 (0.02)
LMP	0.00 (0.00)	− 0.06 (0.08)	− 0.39 (0.08)	− 0.17 (0.10)	

The genetic (co)variance available for selection was obtained for all traits based on Eq. (13). The obtained posterior mean of genetic (co)variance available for selection and the genetic correlations among traits were identical to the results presented in Tables 2 and 3 and are therefore not shown. For FCR, posterior means and PSD of genetic variance available for selection and of genetic correlations with other traits of interest are in Table 4. Posterior means of the genetic correlations of FCR with the two ${\text{RFI}}$ definitions were large and positive. The posterior means of the genetic correlation was negative and low between FCR and ADG (− 0.07) and negative and moderate between FCR and LMP (− 0.40).

**Table 4:** Posterior means of available genetic variance for feed conversion ratio (FCR) and genetic correlations with genetic and phenotypic residual feed intake (**RFI**_**G**_ and **RFI**_**P**_, respectively), average daily feed intake (ADFI), average daily gain (ADG), and lean meat percentage (LMP), with posterior standard deviations in parentheses

	Genetic variance	Genetic correlations				
		RFI_G_	RFI_P_	ADFI	ADG	LMP
FCR	0.010 (0.002)	0.89 (0.04)	0.82 (0.03)	0.52 (0.08)	− 0.07 (0.10)	− 0.40 (0.08)

### Genetic trends

**Fig. 1:**
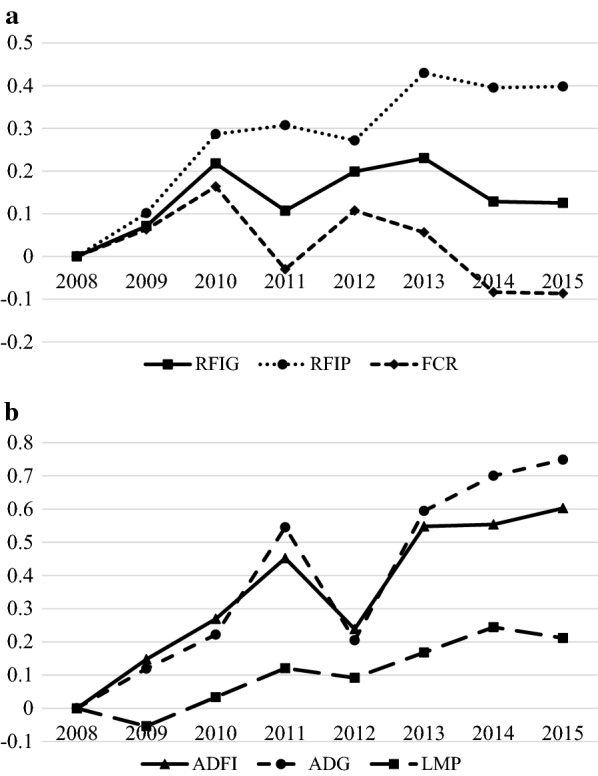
Posterior means of genetic trends for genetic and phenotypic residual feed intake (**RFI**_**G**_ and **RFI**_**P**_, respectively), feed conversion ratio (FCR), average daily feed intake (ADFI), average daily gain (ADG), and lean meat percentage (LMP). All lines for each trait were forced through the same origin in 2008 to make trends comparable across lines. All traits were scaled to the standard deviation of their estimated breeding values to make vertical axes comparable across traits

Posterior means of genetic trends of the traits of interest are presented in Fig. 1. Posterior means of the genetic trend for ${\text{RFI}}_{\text{G}}$ and FCR had a similar pattern but the trend for ${\text{RFI}}_{\text{G}}$ was less favorable. Nonetheless, ${\text{RFI}}_{\text{P}}$ did not follow the trends of ${\text{RFI}}_{\text{G}}$ and FCR. Posterior means of the genetic trend of ADG and ADFI had similar patterns. In addition, the genetic trend for LMP was similar to those for ADG and ADFI. In general, genetic trends indicated improved production traits and FCR. In contrast, both ${\text{RFI}}$ definitions tended to increase, indicating deteriorating partial feed efficiency conditional on production traits.

### Genetic superiority of the selected group

The posterior mean of the direct and correlated superiority of the selected groups under various selection scenarios are in Table 5. Since FCR is a ratio trait in which the numerator should be reduced relative to the denominator, a favorable response to selection is negative. For ${\text{RFI}}$, a negative selection response is also favorable, since the goal is to reduce the proportion of feed intake that is independent of the energy requirements for growth and maintenance. Direct selection on ${\text{RFI}}_{\text{G}}$ resulted in a correlated response of − 0.151 kg/d in ADFI, without altering ADG and LMP. However, direct selection for FCR resulted in a correlated response of − 0.176 kg/d in ADFI, with a 0.978% increase in LMP. Furthermore, direct selection on ${\text{RFI}}_{\text{P}}$ not only reduced ADFI by 0.233 kg/d but also had an unfavorable effect, namely, a 0.035 kg/d reduction in ADG.

**Table 5:** Posterior means of direct (bold figures) and correlated (non-bold figures in a row) additive genetic superiorities of the selected group when the top 10% of the population is selected for single trait selection on feed efficiency or production traits (by row), with posterior standard deviations in parentheses

Scenario^a^	Genetic superiority of the selected group					
	RFI_G_, kg/d	RFI_P_, kg/d	FCR, kg/kg	ADFI, kg/d	ADG, kg/d	LMP, %
RFI_G_	− **0.161 (0.013)**	− 0.158 (0.013)	− 0.155 (0.013)	− 0.151 (0.017)	0.004 (0.004)	0.015 (0.100)
RFI_P_	− 0.149 (0.016)	− **0.170 (0.011)**	− 0.147 (0.014)	− 0.233 (0.020)	− 0.035 (0.010)	0.160 (0.161)
FCR	− 0.144 (0.015)	− 0.145 (0.013)	− **0.174 (0.013)**	− 0.176 (0.025)	0.002 (0.011)	0.978 (0.183)
ADFI	− 0.080 (0.015)	− 0.130 (0.012)	− 0.095 (0.016)	− **0.305 (0.016)**	− 0.088 (0.008)	0.923 (0.159)
ADG	0.002 (0.007)	0.063 (0.016)	− 0.010 (0.018)	0.257 (0.022)	**0.110 (0.007)**	− 0.386 (0.188)
LMP	− 0.002 (0.007)	− 0.013 (0.012)	− 0.072 (0.015)	− 0.123 (0.022)	− 0.020 (0.009)	**2.263 (0.105)**

^a^${\text{RFI}}_{\text{G}}$ = residual feed intake estimated from partial genetic coefficients; ${\text{RFI}}_{\text{P}}$ = residual feed intake estimated from partial phenotypic coefficients; FCR = feed conversion ratio; ADFI = average daily feed intake during test period; ADG = average daily gain during test period; LMP = lean meat percentage at end of the test

## Discussion

In this study, a Bayesian method for estimating genetic parameters for ${\text{RFI}}$ and FCR in farm animals is presented that properly accounts for non-normal distributions of ratio traits. Analyses conducted by Kennedy et al. [5] to derive genetic and phenotypic RFI were extended to Bayesian analysis. Here, we present a Bayesian analysis of FCR without resorting to approximations resulting from unknown distributional properties of a ratio trait and its component traits, which causes the genetic parameters of FCR not to be directly estimable. Instead, we developed a posterior multivariate distribution of additive genetic (co)variance available for selection. The example shows that inference based on this measure is very similar to estimates of additive genetic (co)variance in the population and can, therefore, also be used to investigate the posterior distribution of additive genetic variance in the ratio trait of FCR. Finally, we estimate the posterior distribution of the genetic superiority of the selected group when selection is based on various definitions of feed efficiency or production traits.

### Bayesian method of predicting breeding values for feed efficiency

In this study, a Bayesian approach was used to derive a posterior distribution of all parameters of interest, which enables computation of the probabilities that the parameter lies between specified values. The Bayesian method integrates over all unknown model parameters, including “fixed” and random effects, and properly handles ratio traits that do not have standard distributions.

#### Derivation of RFI

Residual feed intake is a partial measure of feed efficiency, for which the average components of feed efficiency related to production and maintenance are excluded, and which is obtained through the conditional distribution of feed intake to production traits and metabolic body weight. Many studies have used linear regression of the phenotype of feed intake onto phenotypes of the production traits, e.g., Mrode and Kennedy [17]. Some studies took the above approach one step further by using adjusted production traits values, accounting for the systematic effects that influence these traits with the aim of obtaining a more accurate estimation of ${\text{RFI}}$ parameters, e.g., Cai et al. [18] and Shirali et al. [19]. Some studies estimated partial regression coefficients for production traits first and then adjusted the phenotype of feed intake for production traits using the obtained coefficients, e.g., Saintilan et al. [20]. This approach is time-consuming and does not consider the systematic effects of production traits. However, ${\text{RFI}}$ is originally defined as a residual effect from regression models that account for BW growth and gain by Koch et al. [4]. The methods for obtaining genetic or phenotypic ${\text{RFI}}$ that use genetic or phenotypic (co)variance matrices from a multi-trait model were presented by Kennedy et al. [5]. Phenotypic derivation of ${\text{RFI}}$ ensures that the phenotypic correlation between ${\text{RFI}}$ and its component traits of production traits are zero, but the genetic correlations can still be non-zero, as shown by Kennedy et al. [5]. The non-zero genetic correlation of ${\text{RFI}}_{\text{P}}$ with production traits is due to partial phenotypic regression coefficients, which result in a genetic correlation between ${\text{RFI}}_{\text{P}}$ and production traits. This genetic correlation is related to genetic and environmental covariances between feed intake and production traits, as well as the heritability of production traits, as shown by Kennedy et al. [5]. To obtain a genetic ${\text{RFI}}$, partial regression coefficients must be obtained from the genetic (co)variance matrix, which ensures that ${\text{RFI}}$ is genetically independent of production traits. However, this can result in non-zero phenotypic correlations of genetic ${\text{RFI}}$ with production traits, i.e. *cov*(*y*_*RFI*_, *y*_*p*_) = *cov*(*y*_*FI*_, *y*_*p*_) − *b*_*g*_*var*(*y*_*p*_), which is equal to *cov*(*e*_*FI*_, *e*_*p*_) − *cov*(*g*_*FI*_, *g*_*p*_)(1 − *h*_*p*_^2^)/*h*_*p*_^2^), as also shown by Kennedy et al. [5]. Variation in maintenance requirements that are predicted from differences in metabolic body weight have not been significantly related to variation in feed consumption in pigs, as tested by Cai et al. [18], Shirali et al. [19], and the current study. This could be due to a relatively set body weight test period in pig breeding. Nonetheless, in future studies and in selection practices, the effect of metabolic body weight on the variation of feed intake should be tested since the results can vary depending on the species and breeding programs.

#### Derivation of FCR

Traditionally, FCR is derived by dividing the phenotype of feed intake by BW gain. This definition ignores the fixed and environmental effects that influence the component traits of the ratio trait. The Bayesian analysis presented here considers the uncertainties in the fixed effects and avoids approximations due to unknown distributional properties of a ratio trait and its component traits.

### Genetic parameters for feed efficiency and production traits

#### Genetic background of RFI

The current study shows substantial genetic variance in ${\text{RFI}}$, which illustrates the possibility of selection for this trait in commercial breeding programs. Genetic ${\text{RFI}}$ showed a low posterior mean of heritability, lower than for phenotypic ${\text{RFI}}$, which is as expected, with few exceptions, as explained by Kennedy et al. [5] because the genetic variance of phenotypic ${\text{RFI}}$ is influenced by residual covariance between the component traits of feed intake and production traits. The posterior means of heritability estimates for genetic and phenotypic ${\text{RFI}}$ were within the range of values (0.10–0.47) reported in the literature [6, 20, 21].

The percentage of genetic variance in ADFI that was explained by genetic ${\text{RFI}}$ had a posterior mean of 26%, with a PSD of 6%. Thus, considerable genetic variance in ADFI is not due to production traits (ADG and LMP). Shirali et al. [6] reported that the proportion of genetic variance in feed intake explained by genetic ${\text{RFI}}$ ranged from 17 to 26% for three Danish pig breeds. Cai et al. [18] and Shirali et al. [19] reported that 34 and 33% of the phenotypic variation in feed intake is due to phenotypic ${\text{RFI}}$ in Yorkshire and crossbred pigs, respectively.

The considerably lower posterior mean of heritability for genetic ${\text{RFI}}$ compared to ADFI is due to high genetic correlations between ADFI and production traits and to genetic correlations between traits being higher than environmental correlations. Nevertheless, feed intake records provide valuable information on feed efficiency over and above that provided by the production traits ADG and LMP.

#### Genetic background of production traits

Posterior means of heritability and genetic variance for ADG and LMP obtained here were larger than those for Danish Duroc pigs that were reported in Shirali et al. [6]. The larger posterior mean of the heritability for ADFI compared to ADG is in agreement with results of Shirali et al. [6] for three diverse Danish pig breeds and of Saintilan et al. [20] for French Landrace and Large White sire and dam lines. Posterior means of genetic correlations between ADFI and ADG were larger than the corresponding phenotypic correlations, which is in agreement with Shirali et al. [6].

#### Genetic correlation between feed efficiency and production traits

The substantial deviation from 1 of the posterior mean of the genetic correlation between genetic and phenotypic ${\text{RFI}}$ indicates different selection outcomes when selecting on these respective traits. The posterior mean of the genetic correlation between phenotypic ${\text{RFI}}$ and ADFI was in the upper range of values (0.48–0.72) reported by Saintilan et al. [20] and in the range of values (0.70–0.88) reported by Do et al. [22]. The posterior mean of the genetic correlation between phenotypic ${\text{RFI}}$ and ADG was larger than the genetic correlations reported by Saintilan et al. [20] (− 0.05 to 0.16) and Do et al. [22] (0.02–0.20). Dekkers and Gilbert [23] showed genetic correlations of 0.18 and 0.24 for phenotypic ${\text{RFI}}$ with growth rate and backfat thickness, respectively in a divergent selection experiment for phenotypic ${\text{RFI}}$ at Iowa State University, while Gilbert et al. [24] reported genetic correlations of − 0.07 and 0.14 for phenotypic ${\text{RFI}}$ with growth rate and carcass lean meat content, respectively, in similar experiments at INRA. Kennedy et al. [5] showed that phenotypic ${\text{RFI}}$ and production traits are genetically independent when the heritabilities of feed intake and production traits are equal and their genetic and environmental correlations are equal. The partial phenotypic coefficient ensures that phenotypic ${\text{RFI}}$ is phenotypically independent of production traits, explaining the positive moderate and negative low posterior means of genetic correlations of phenotypic ${\text{RFI}}$ with ADG and LMP, respectively.

The Bayesian method provides a method to investigate the variance and covariances of the ratio trait of FCR without resorting to approximations. The posterior mean of the genetic variance for FCR was substantially lower than the estimates of 0.014 to 0.027 reported by Do et al. [22]. The posterior means of genetic correlations between FCR and different definitions of ${\text{RFI}}$ deviated significantly from 1. Saintilan et al. [20] reported genetic correlations of 0.53 to 0.85 between FCR and phenotypic ${\text{RFI}}$, and Do et al. [22] reported values of 0.87 to 0.88, which are in line with our results. The posterior mean of the genetic correlation between FCR and ADFI was in the middle range of the values reported by Saintilan et al. [20] (0.20–0.88) and by Do et al. [22] (0.43–0.74). The posterior mean of the genetic correlation between FCR and ADG was in the lower range of values reported by Saintilan et al. [20] (− 0.09 to − 0.51) and was in the range of those by Do et al. [22] (− 0.38 to 0.26). The posterior mean of the genetic correlation between FCR and LMP was larger than the estimates of − 0.15 to 0.03 between FCR and lean meat content in Saintilan et al. [20] and of − 0.36 to 0.34 between FCR and backfat thickness in Do et al. [22].

#### Genetic trends

The genetic improvement in FCR can be explained by genetic trends for feed intake and production traits. Using realized genetic trends, on the basis of units of genetic standard deviation, for production and feed efficiency traits in four PIC pig lines from 2001 to 2011, Knap and Wang [25] reported that genetic improvement for RFI is slower than for FCR and that the genetic trend of FCR is the result of genetic trends in ADG and ADFI. This was also observed in our study, possibly because the genetic trend of FCR is influenced by production traits, while the genetic trend of ${\text{RFI}}$ is for the proportion of feed intake that is independent of production traits, which has not been under direct selection. In fact, FCR improved over the period studied, because ADG increased more than ADFI. Efficiency defined in terms of genetic ${\text{RFI}}$ deteriorated over the examined period.

### Bayesian estimates of direct and correlated responses to selection

#### Additive genetic (co)variance available for selection

Applying the Bayesian approach to the data yields a marginal posterior distribution of breeding values for the analyzed traits and for any function of them, from which inferences can be made that take the inaccuracy of the knowledge of variances into account. We derived the marginal posterior distribution of additive genetic variance available for the selection of traits of interest from the population under study, considering the genetic trend in each year in a multivariate setting. This is an extension of Sorensen et al. [10], who conditioned additive genetic variance for the genetic trend in a Bayesian setting for a univariate model.

#### Bayesian estimation of genetic superiority of the selected group

The current study presents a new approach using Bayesian inference to examine various selection criteria for feed efficiency either as a linear (${\text{RFI}}$) or ratio (FCR) trait in breeding programs. The method yields a marginal posterior distribution of the average response to selection of selected groups, which can be viewed as a weighted average of an infinite number of conditional distributions. The method also allows PSD of the expected response to selection to be derived easily.

Gunsett [3] also showed unexpected selection pressure on component traits of ratio traits and that a ratio trait is not a normally distributed variable, as it is a ratio of two normally distributed variables. Therefore, expected genetic gain from truncation selection on FCR is difficult to compute using selection index principles for normally distributed variables. Gunsett [3] observed that direct selection for ratio traits places a large proportion of the selection pressure on reducing the numerator, while using a linear index of component traits of ratio traits would allocate more weight to increasing the denominator. Based on a simulation study, Zetouni et al. [26] reported that direct selection against the methane-to-milk production ratio trait increased the denominator and the numerator, while multi-trait selection could result in higher genetic gain and a simultaneous reduction in methane emission.

The Bayesian approach allows identification, with high accuracy, of the possible outcomes of any combination of single or multi-trait selection on feed efficiency and/or traits in the breeding program. Bayesian analysis is useful to study the design of selection experiments, since it allows a variety of designs, and allows comparison of their efficiency in retrieving accurate marginal posterior distributions of parameters of interest [9]. An advantage of the proposed Bayesian approach is that the posterior distribution of direct and correlated responses to selection can be obtained and used to make probability statements on expected response to selection as well as other parameters of interest. It should also be noted that the principles outlined in this study have much broader applications beyond FCR, as they apply to any trait that is defined as a non-linear function of other traits.

Bayesian analysis suggests that direct selection against genetic ${\text{RFI}}$ does not have a correlated response on production traits in the breeding program, since the model ensured zero genetic correlations between these traits. The presence of a correlated response on production traits from direct selection against phenotypic ${\text{RFI}}$ is due to the genetic correlation between these traits, which is due to the use of phenotypic partial regression coefficients that ensure that the phenotypic correlations between phenotypic ${\text{RFI}}$ and production traits are zero. Kennedy et al. [5] observed that response to selection on genetic ${\text{RFI}}$ increases if the genetic correlation between feed intake and production is low or the heritability of feed intake is high or higher than the heritability of the production trait. Young and Dekkers [27] and Gilbert et al. [24] showed that selection for phenotypic ${\text{RFI}}$ resulted in correlated responses in other traits, with a reduction in FCR, backfat thickness, and feed intake in experimental selection lines of purebred Yorkshire and Large White pigs. Young and Dekkers [27] showed that eight generations of selection against phenotypic ${\text{RFI}}$ in Yorkshire pigs decreased ${\text{RFI}}$ by 241 g/d, feed intake by 376 g/d, growth rate by 79 g/d, FCR by 2.2 g/g, and back fat thickness by 2.5 mm compared to a line selected as control line for five generations and thereafter for high RFI. Similar results were obtained in an experiment at INRA with the low ${\text{RFI}}$ line having lower ${\text{RFI}}$, feed intake, growth rate, and backfat thickness than the high RFI line reported by Gilbert et al. [24]. Kennedy et al. [5] observed that response to selection on genetic ${\text{RFI}}$ is less than or equal to the response to phenotypic ${\text{RFI}}$ because selection for phenotypic ${\text{RFI}}$ results in a reduction of the proportion of feed intake used for production traits. Genetic ${\text{RFI}}$ is a product of genetic parameters of the traits that are involved in the calculations of ${\text{RFI}}$. Therefore, accurate estimation of genetic parameters of the traits involved in the calculation of ${\text{RFI}}$ is necessary to maximize response to selection. Our proposed Bayesian approach maximizes gain by averaging over the posterior distribution of variance components for the traits involved.

Selection against the ratio trait of FCR results in unexpected selection pressure on feed intake and production traits (e.g., LMP) in the breeding program. This disproportionate selection pressure on component traits can be explained by genetic correlations between ADFI, ADG, and LMP and their heritabilities. A large reduction in ADFI, which is the numerator of FCR, may be due to the heritability of ADFI being higher than that of ADG, in addition to a large positive posterior mean of the genetic correlation between these traits. The low posterior mean of the genetic correlation between FCR and ADG indicates a smaller change in ADG due to selection for FCR, while a large negative posterior mean of the genetic correlation between FCR and LMP explains the indirect genetic response from selection against FCR. The substantial reduction in ADFI through direct selection on FCR could also be due to a correlated response on LMP, since increased lean meat growth is one of the underlying biological reasons for improved FCR. Therefore, selection for FCR is not an efficient strategy because, first the improvement in this trait can be due to improvement in lean meat growth rather than improvement in efficiency of nutrient utilization per se; and second the relative improvements can change over generations as the means of the underlying trait change. Shirali et al. [6] reported low negative genetic correlations between feed intake and BW gain in Danish Duroc pigs, while for Danish Landrace and Yorkshire pigs, they were high negative in the range reported here. In addition, genetic correlations of ADG in the 30 to 100 kg BW test period with LMP at the end of the test on Danish pigs were lower [6] than the posterior mean of the correlation between ADG and LMP in our study. Differences in genetic parameters of feed efficiency traits between breeds or breeding programs can result in differences in the outcome of the selection for a ratio trait such as FCR.

Gunsett [3] reported that selection intensity for a ratio trait influences the relative distribution of response in the component traits when selection intensity increases resulting in more selection pressure on reduction of the numerator of the ratio trait. In our study, a change in selection intensity did not alter the relative responses for linear and ratio feed efficiency traits and for production traits, providing a robust conclusion for the effects of selection on different traits.

In a selection index context, single-trait selection against genetic ${\text{RFI}}$ is equivalent to selection on an index for feed intake that maintains production constant and considers no other traits in the breeding program. Luiting et al. [28] showed that joint selection on ${\text{RFI}}$ and production traits is equivalent to joint selection on a selection index of feed intake and production traits. Furthermore, Kennedy et al. [5] showed that selection on an index that includes either genetic or phenotypic ${\text{RFI}}$, or ADFI, would result in the same responses to selection, provided that the corresponding economic weights are changed in the breeding goal. However, this is only possible by estimating proper economic values when using phenotypic ${\text{RFI}}$ or ADFI. If the economic value of ADFI, ADG, and LMP are known, phenotypic and genetic (co)variances are needed to derive the corresponding economic weight for RFI. Genetic RFI can be used in a breeding program because it is easy to communicate to farmers/breeders since it expresses net feed efficiency rather than efficiency achieved by improvement on production traits. Furthermore, genetic ${\text{RFI}}$ can be suitable in selection experiments to provide insight into the biological basis of feed efficiency and variation in feed intake independent of production and maintenance requirements.

## Conclusions

A Bayesian procedure for analysis of response to selection on linear versus ratio traits was developed and applied to feed efficiency in pigs. The Bayesian methodology allowed prediction of breeding values for ratio and linear definitions of feed efficiency from a multi-variate model for the traits measured. The Bayesian method allowed prediction of breeding values for FCR without the need for approximations. Posterior means of genetic parameters indicated that the traits were influenced by genetics and that genetic improvement through selection was possible. Direct selection against FCR or ${\text{RFI}}_{\text{P}}$ resulted in disproportional selection on production traits. Direct selection against FCR results in unexpected selection pressure on its component traits and on LMP. However, direct selection against genetic ${\text{RFI}}$ allows for selection on the proportion of ADFI that is independent of production. In addition, since there is no genetic correlation between genetic ${\text{RFI}}$ and other production traits in the breeding program, an EBV for ${\text{RFI}}$ that is independent of production traits is easier to communicate to farmers/advisors than a breeding value for ADFI that is strongly influenced by production traits such as ADG and LMP.
